# Effect of an Education Program on Nutrition Knowledge, Attitudes toward Nutrition, Diet Quality, Lifestyle, and Body Composition in Polish Teenagers. The ABC of Healthy Eating Project: Design, Protocol, and Methodology

**DOI:** 10.3390/nu10101439

**Published:** 2018-10-05

**Authors:** Jadwiga Hamulka, Lidia Wadolowska, Monika Hoffmann, Joanna Kowalkowska, Krystyna Gutkowska

**Affiliations:** 1 Department of Human Nutrition, Faculty of Human Nutrition and Consumer Sciences, Warsaw University of Life Science (SGGW-WULS), 159C Nowoursynowska Street, 02-787 Warsaw, Poland; 2 Department of Human Nutrition, Faculty of Food Sciences, University of Warmia and Mazury in Olsztyn, 45F Sloneczna Street, 10-718 Olsztyn, Poland; lidia.wadolowska@uwm.edu.pl (L.W.); joanna.kowalkowska@uwm.edu.pl (J.K.); 3 Department of Functional Food, Ecological Food and Commodities, Faculty of Human Nutrition and Consumer Sciences, Warsaw University of Life Science (SGGW-WULS), 159C Nowoursynowska Street, 02-787 Warsaw, Poland; monika_hoffmann@sggw.pl; 4 Department of Organization and Consumption Economics, Faculty of Human Nutrition and Consumer Sciences, Warsaw University of Life Science (SGGW-WULS), 159C Nowoursynowska Street, 02-787 Warsaw, Poland; krystyna_gutkowska@sggw.pl

**Keywords:** adolescents, dietary habits, education program, hand grip strength, nutrition knowledge, obesity, physical activity, public health intervention, sedentary lifestyle, three-factor eating questionnaire

## Abstract

To increase teenagers’ nutrition knowledge is an important target and has the potential to improve their dietary habits and lifestyle while reducing incidences of obesity-related non-communicable diseases throughout the whole lifespan. This study protocol presents the general approach and details of an assessment of nutritional knowledge, attitudes toward nutrition, diet quality, lifestyle and body composition that have been used to comprehensively evaluate the cross-behavioral patterns covering dietary and lifestyle behaviors in Polish teenagers. The study was designed in two paths as: a cross-sectional study (covering 1569 students) and an education-based intervention study (464 students) with a 9-month follow-up. We describe a short form of the food frequency questionnaire (SF-FFQ4PolishChildren) used to collect data and details of diet-related and lifestyle-related education program, which was developed and implemented by academic researchers involved in the study. We also describe details of the data development and statistical analysis, including multidimensional methods of clustering variables to identify cross-behavioral patterns covering diet and lifestyle. The results of the study will provide evidence-based support for preventive health care to promote normal growth and development of young population and reduce the risk of diet-related diseases in adulthood, by early shaping of adequate dietary and lifestyle behaviors. In the future, well-tailored education programs addressed to teenagers can be created as an important public health action, based on our results.

## 1. Introduction

Overweight and obesity are among the most serious public health challenges of the 21st century [1,2,3]. The prevalence of childhood obesity is increasing in most regions of the world, in both developed and developing countries [4,5]. Recent studies suggest that these trends are gradually stabilizing or have already reached a plateau in some countries [6]; however, the problem remains significant [7,8]. According to the World Health Organization (WHO), in Europe in 2009–2010, on average, one in every three children aged six to nine years was overweight or obese, while among children aged 11 and 15 years the prevalence of overweight and obesity was 11–33% and 10–23%, respectively [9]. It is expected that if the current trend continues, there will be 20% or more obese children and adolescents in over 30 countries around the world in 2025 [3]. Among the European countries, Poland has one of the highest rates of prevalence of overweight in children and adolescents; 12–25% of Polish children and young people are overweight with a tendency toward an increase in the prevalence of both overweight and abdominal obesity [5,10,11,12,13].

Being overweight in early childhood has been shown to increase the chance of being obese in later childhood and also leads to adulthood obesity [2,7,11,14]. It is estimated that around 55% of obese children and 70% of obese adolescents will experience adult obesity [15], which increases their risk of obesity-related non-communicable diseases, such as diabetes, cardiovascular disease, various types of cancer, as well as premature mortality [16,17,18,19]. Therefore, prevention and treatment of childhood obesity is of the utmost importance, given the significant health and social consequences both in the short and long terms [20,21].

Obesity has a multi-factorial and multi-level etiology with many factors such genetic, physiological, environmental, and socioeconomic, including gender, family affluence, and education level [12,22,23,24]. Although genetic and biological factors are important, they cannot fully explain the current global childhood obesity trends. Environmental and societal factors associated with food intake and physical activity should be the primary focus for understanding the macro-level impact on obesity [5,9,11,24]. Rapid changes in ways of spending leisure time, a decline in physical activity, the pressure of peers, and the fashion of using electronic devices and almost unlimited access to food create an ”obesogenic” environment with two main factors affecting the positive energy balance and obesity—sedentary lifestyle and unhealthy diet [10,25,26,27,28]. Food marketing also plays a role by increasing children’s exposure to obesogenic foods because children and adolescents are more susceptible to food marketing than adults [29]. The availability of snack vending machines, bars, and kiosks with snack food and soft drinks in schools was also associated with lower fruits and vegetable consumption, higher intake of calories from total and saturated fat, and a higher body mass index (BMI) z-score [30,31].

As childhood obesity has a multi-factorial background, it should be tackled at multiple levels, including individual, household, school, and social [32,33]. Knowledge and beliefs related to health can improve health behaviours, especially when they are part of a targeted intervention, according to the Integrated Theory of Health Behaviour Change [34,35]. Improving nutrition knowledge can be assumed to contribute to the enhancement of dietary habits and food choices in those exposed to education-based intervention [32,33,36].

Since poor dietary habits tend to be carried over from childhood to adulthood [37], childhood and adolescence are periods when good nutritional diet quality is important to establish healthy dietary behaviours. The family environment is a well-known factor influencing the food consumption of children, especially in the early years of life. The influence of peer environment, mass-media, celebrities and schools (due to school nutrition programs) increases rapidly during adolescence [38]. In this period, an awareness of one’s own sexuality and gender differences with regard to diet and health as well as physical abilities arise. The precise indication of the age at which changes start in the perception of oneself as an independent individual and food consumer is difficult, but the age of 10 (12) years old (depending on gender) is considered a breakthrough [39]. Thus, early adolescence may be the last moment for implementing sustained healthy school culture and nutrition education addressed to young people before determining their relatively stable eating habits.

Schools are considered a primary setting for implementing education programs—they have the unique potential of involving a wide population of children and teenagers. A health education curriculum that highlights the importance of nutrition and physical activity can help students adopt and maintain healthy lifestyles regarding eating and physical activity [40,41]. Across the world, there is a wide range of nutrition education initiatives addressed to school children, with the participation of schools, government and health promotion agencies delivering knowledge about diet components [42] and specific education to prevent or manage dietary-related and lifestyle-related diseases.

In Poland, multi-disciplinary programs covering the implementation of multi-component activities are mainly directed at preschool children (e.g., ToyBox-study, Program Eating Healthy, Growing Healthy) [43,44,45]. While programs conducted in schools are focused on single topics, e.g., culinary workshops, nutrition education classes or physical activity education (POL-HEALTH; “Zachowaj Równowagę”), there is a lack of a comprehensive approach with well-documented scientific research [46,47,48]. There have been relatively few comprehensive educational programs in Poland, which have been combined with a scientific assessment of nutrition knowledge, dietary habits and lifestyle. Therefore, our project is aimed at filling the gap, with the potential that our research conducted on Polish teenagers can be interpreted in a wider scope for other European teenagers living in similar conditions.

The aim of the current paper is to describe the design, methodology and approach of the study which was based on “ABC of Healthy Eating” project, and also to present the overall characteristics of the study sample as assessed at baseline, prior to the implementation of a diet-related and lifestyle-related education program.

### 1.1. Research Objective and Hypothesis

Our study is focused on teenagers aged 11–13 years, taking into account that early adolescence may be the last moment for implementing nutrition-related and lifestyle-related education before determining their relatively stable habits. We hypothesized that education focused on improving teenagers’ dietary and lifestyle behaviours can increase the level of nutrition knowledge and positively influence attitudes toward nutrition, dietary behaviours, lifestyle, and body composition. It was assumed that education should be multi-component and use various forms of education tailored to teenagers’ needs and their perceptions. However, the education effect can decrease over time and the rate of this change depends on the initial level of nutrition knowledge and socio-demographic factors. 

There were two aims of the study:

- To assess the short- and medium-term effect of nutrition-related and lifestyle-related education program on nutrition knowledge, attitudes toward nutrition, diet quality, lifestyle, and body composition of Polish teenagers in a socio-demographic context.
- To determine the association between teenagers’ nutrition knowledge, attitudes toward nutrition, dietary and lifestyle behaviours, body composition, and socio-demographic factors.

### 1.2. Study Design 

Data related to the study were collected as a part of:

- A national multicenter “ABC of Healthy Eating” project—the 1st edition in 2015–2016 (“ABC of Healthy Nutrition”) and the 2nd edition in 2016 (“ABC of Kids’ Nutrition”) (Figure S1).
- The own research of academic centers involved in the study, carried out in parallel with those two editions of the project.

The study had a wider scope and went beyond the activities carried out under the “ABC of Healthy Eating” project.

**Figure 1:**
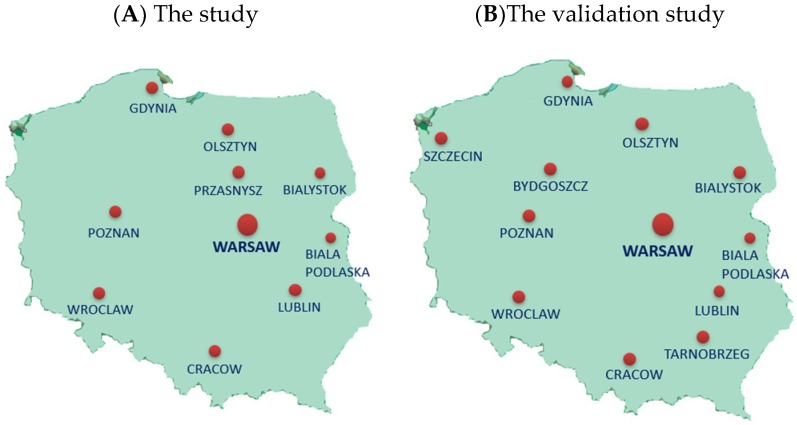
The location of academic centers involved in the study (**A**) or the validation study (**B**). **A**: Universities (cities) involved in the study: Warsaw University of Life Sciences, WULS-SGGW (Warsaw, Przasnysz); Gdynia Maritime University (Gdynia); Medical University in Bialystok (Bialystok); Josef Pilsudski University of Physical Education in Warsaw—The branch in Biala Podlaskiej (Biala Podlaska); University of Agriculture in Krakow (Cracow); University of Life Sciences in Lublin (Lublin); University of Life Sciences in Poznan (Poznan); University of Warmia and Mazury in Olsztyn (Olsztyn); Wroclaw University of Environmental and Life Sciences (Wroclaw). **B**: Universities (cities) involved in the validation study: Warsaw University of Life Sciences, WULS-SGGW (Warsaw, Tarnobrzeg); Gdynia Maritime University (Gdynia); Medical University in Bialystok (Bialystok); Josef Pilsudski University of Physical Education in Warsaw—The branch in Biala Podlaskiej (Biala Podlaska); University of Agriculture in Krakow (Cracow); University of Life Sciences in Lublin (Lublin); University of Life Sciences in Poznan (Poznan); University of Warmia and Mazury in Olsztyn (Olsztyn); Wroclaw University of Environmental and Life Sciences (Wroclaw); University of Economy (Bydgoszcz); West Pomeranian University of Technology (Szczecin).

The study was carried out on students of selected elementary schools from urban, sub-urban, and rural areas covering the entire territory of Poland. The schools (not randomly selected) were located a convenient distance from nine Polish universities in ten locations. In 2015–2016, the study was carried out in schools located near following cities: Gdynia, Cracow, Lublin, Olsztyn, Poznan, Przasnysz, Warsaw, Wroclaw, and in 2016, in schools located near: Biala Podlaska, Bialystok, Gdynia, Cracow, Lublin, Olsztyn, Warsaw, and Wroclaw (Figure 1A).

In April–May 2015 the research concept and a short form of the food frequency questionnaire (SF-FFQ4PolishChildren) dedicated to school children were developed. The main study was preceded by pilot studies (in May 2015) in which the research procedure and tools were verified in all centers, covering the 200 subjects above. Data collection for the main study started in June 2015. The data were collected and measurements were taken by well-trained researchers. The study started at the same time in all centers involved in the study.

The study was carried out in two paths: as an education-based intervention design and a cross-sectional design. The study is reported according to the guidelines of reporting of observational and intervention studies of public health in nutritional epidemiology [49,50,51].

### 1.3. The Education-Based Intervention Study 

Two groups were established: under intervention (educated group) and without intervention (control group) by accidental allocation of school classes to the educated or control group. In the educated group, a diet-related and lifestyle-related education program lasting three weeks was implemented. The program consisted of 5 topics, each topic included various forms of education from fun to “scientific” cognition (Table 1). Each topic lasted approx. 180 min (4 h of school lessons) and was run by a minimum of 3–4 researchers. The program was developed and implemented by academic researchers involved in the study. School teachers were not involved in the education program, they were only present during educational activities. Apart from the study, students from both educated and control groups took part in regular school activities containing some content related to nutrition and a healthy lifestyle.

The overall content of the intervention-based educational study is presented in Table 2. The data was collected 4 times: (i) before education (at baseline), (ii) 3 weeks from baseline at the end of the education program (in the educated group only), (iii) after 3 months (±2 weeks; 3-month follow-up) to measure the short-term effect of education, (iv) after 9 months (±2 weeks; 9-month follow-up) to measure the medium-term effect of education.

### 1.4. The Cross-Sectional Study 

**Figure 2:**
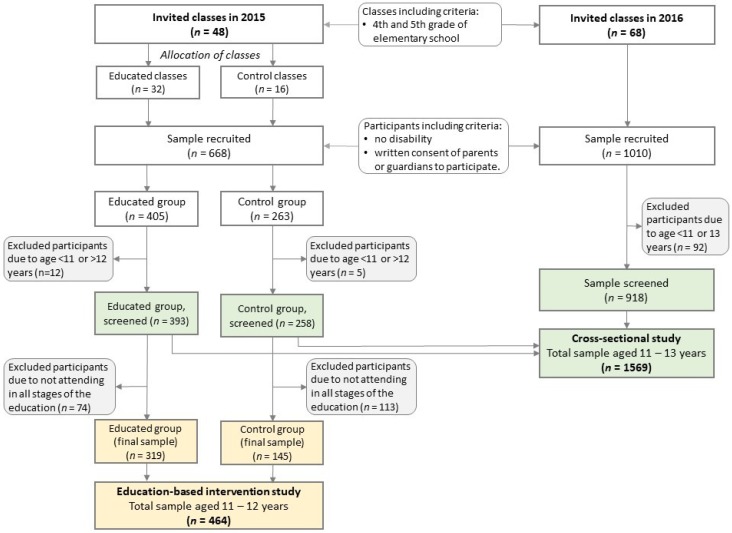
Flow chart of sample collection and study design.

In this study design, pooled data were used (see Figure 2 in Section 2.2) covering the data collected in June 2015 within the intervention-based educational study and June 2016 within the 2nd edition of the project ‘ABC of Kids’ Nutrition’ (Table 3). In this database, all data were collected before education program in those participants who have been subjected to the education, i.e., all participants from educated group in 2015 (at baseline) and all participants in 2016. In the 2nd edition of the project, no effect of education was measured and no control group was established.

## 2. Materials and Methods 

### 2.1. Ethical Aspects

The project followed the ethical standards recognized by the Declaration of Helsinki and was approved by the Bioethics Committee of the Faculty of Medical Sciences, University of Warmia and Mazury in Olsztyn on 17 June 2010, Resolution No. 20/2010. The study was explained to the participants before the start (verbally and by leaflets), to the pupils at the school lessons and to the parents or legal guardians during the meetings. Parental or legal guardians’ written informed consent was obtained for the participation of their children. The consent form was distributed for parents or legal guardians during the meetings. All data regarding the study were collected at the time designated for school classes instead of regular school lessons. Participants who refused to participate in the study attended other school activities.

### 2.2. Participant Selection

A convenient sample selection was applied in both the education-based intervention study and the cross-sectional study. A school class was the smallest unit in the sample selection. All students from a given class were invited to take part in the study. Recruitment was conducted in selected elementary schools (see in Section 1.2). Table 4 presents the criteria of schools and participants’ inclusion or exclusion. 

Students from 4th- and 5th-grade classes were invited to attend. The expected ages of the students were 11–12 years in 2015 and 11–13 years in 2016 due to changes in the Polish law concerning the age of starting education by children (obligatory or optional starting from six years). We decided to start recruitment based on school classes because students were subject to the same school education and would be at a similar stage of development. In 2015, 48 classes were invited (educated group/control group: 32/16 classes) and in 2016, 68 classes were invited (Figure 2). 

In the education-based intervention study, 668 students (educated/control: 405/263) were initially recruited. Due to age (12/5) or not attending all stages of the study (74/113), 208 participants were excluded from the analyses. Finally, the study included 464 teenagers (319/145) with a baseline age of 11–12 years, including 216 boys (46.6%) and 248 girls (53.4%).

In the cross-sectional study, 1,678 students were initially recruited. Due to age (in 2015/in 2016: 17/92), 109 participants were excluded from analyses. Finally, the study included 1569 teenagers aged 11–13 years, 760 boys (48.4%) and 809 girls (51.6%).

### 2.3. Questionnaire for Data Collection 

Data related to nutrition knowledge, attitudes toward nutrition, diet, lifestyle and socio-demographic factors were collected with 50-item, a short form of food frequency questionnaire (SF-FFQ4PolishChildren) developed by Kowalkowska, Wadolowska, and Hamulka for the “ABC of Healthy Eating” project (Tables S1 and S2).

A validation procedure of the short form of food frequency questionnaire (FFQ) was carried out, regardless of this study, in cooperation with academic researchers from 12 universities covering the entire territory of Poland (data not published, paper in preparation) (Figure 1B). In brief, the internal compatibility of the questionnaire was tested by 630 teenagers, aged 11–15. The questionnaire was completed by teenagers twice (test and retest after 2 weeks). Fleiss’ Kappa for breakfast consumption was 0.54, school meal consumption was 0.53, pro-Healthy Diet Index categories (see in this section below) was 0.44 and the non-Healthy Diet Index categories was 0.35, Family Affluence Scale items were 0.69 to 0.83, nutrition knowledge level was 0.36, screen time categories was 0.46 and physical activity level was 0.52. Compatible classification of subjects (into the same category in test and retest) was for breakfast, 77.3%; school meal, 79.8%; pro-Healthy Diet Index, 75.8%; non-Healthy Diet Index, 92.0%; Family Affluence Scale items, 83.7% to 95.8%; nutrition knowledge level, 72.7%; screen time, 64.8%; total physical activity level, 74.8%. The internal compatibility of the short form of FFQ dedicated for teenagers was considered acceptable to good.

### 2.4. Nutrition Knowledge 

The nutrition knowledge level was determined based on eighteen questions (Q22 to Q39; Table S1). Participants were asked about nutrition based on questions developed by Whati [53] and adapted to Polish conditions and education based on the Polish Food Guide Pyramid [52]. Correct answers were scored with 1 point and wrong or “don’t know” answers and missing data were scored with 0 points. Points were summed up for each respondent. Based on tertile distribution (data from the cross-sectional study), the respondents were divided into three nutritional knowledge levels labeled as follows: the lowest (0–4 points), moderately low (5–7 points), and higher (8–18 points).

### 2.5. Attitudes toward Nutrition

Based on a three-factor eating questionnaire (TFEQ13), dedicated to children, attitudes toward nutrition were determined. The questionnaire was adapted and validated to the Polish conditions by Dzielska et al. [54]. Originally, TFEQ13 consisted of 13 statements, but a weak understanding of three statements was found during pilot studies. Therefore, we used a shorter version of the questionnaire (TFEQ10) comprising 10 statements (Q12 to Q21; Table S1. According to Dzielska et al. [54], three subscales, in shortened version, were developed: Emotional Eating (Q12, Q14), Uncontrolled Eating (Q13, Q15, Q16, Q17, Q20), and Cognitive Restraint of Eating (Q18, Q19, Q21). The scores of each subscale were calculated as a sum of points assigned to answers given by respondent. Answers were scored as follows (points):

- Q12 to Q20: respondents could choose one of four answers: “definitely yes” (3 points), “rather yes” (2 points), “rather not” (1 point), “definitely not” (0 points),
- Q21: respondents could choose one of eight points on the graphical scale: 1 or 2 (0 points), 3 or 4 (1 point), 5 or 6 with (2 points), 7 or 8 (3 points).

### 2.6. Diet Quality

The questionnaire was self-administered by teenagers in the classroom and supervised by well-trained researchers. Explanations were given if necessary. The completion of the questionnaire took the teenagers approx. 40 min.

Respondents were asked to specify their usual frequency consumption within the last 12 months for breakfast and school meal and nine food items such as dairy products (e.g., milk, yogurt, cottage cheese, cheese), fish (e.g., baked, smoked, fried, in vinegar, canned), vegetables (e.g., fresh, boiled, baked, stewed), fruit (fresh or frozen), fruit or mixed fruit-veggie juices, fast foods (e.g., chips, pizza, hamburgers), sweetened carbonated drinks (e.g., cola-type, water with syrup, tea-type with sugar), energy drinks, sweets or confectionery (e.g., cookies, sweets, cake, chocolate bars, chocolate).

Breakfast was considered as consuming solid foods with or without beverages at the first eating episode of a day [55,56]. Drinking only beverages was not considered as breakfast. Respondents could choose one from four categories of breakfast consumption (number of days/week): 0/week, 1–3/week, 4–6/week, and 7/week. Some categories have been combined after distribution analysis. Finally, breakfast consumption was analyzed in three categories as follows: every day (7/week), irregular (4–6/week), and skipping (0–3/week).

The school meal was considered as consuming solid foods with or without beverages at the second eating episode of a day while at school as lunch or a so-called second breakfast (more typical in Poland). Drinking beverages only was not considered as a school meal. Respondents could choose one from four categories of school meal consumption (number of days/week): 0/week, 1–2/week, 3–4/week, and 5/week. Some categories were combined after distribution analysis. Finally, school meal consumption was considered in three categories as follows: every day (5/week), irregular (3–4/week), and skipping (0–2/week).

Breakfast and school meal consumption considered together were analyzed in three categories as follows: every day (breakfast: 7/week; school meal: 5/week), skipping (breakfast: 0–3/week; school meal: 0–2/week), and irregular (other frequencies).

For food frequency consumption, respondents could choose one from the following seven categories (converted into daily frequency, times/day): never or almost never (0 times/day), less than once a week (0.06 times/day), once a week (0.14 times/day), 2–4 times/week (0.43 times/day), 5–6 times/week (0.79 times/day), every day (1 time/day), and several times a day (2 times/day).

Two diet quality scores were used as follows: a pro-Healthy Diet Index (pHDI) and a non-Healthy Diet Index (nHDI). Both diet quality scores were established (a priori approach) on the basis of usual food frequency consumption within the 12 last months. The pHDI included four food items as follows: dairy products, fish, vegetables and fruit; the nHDI included four food items as follows: fast foods, sweetened carbonated drinks, energy drinks and sweets or confectionery. Diet quality scores were calculated by summing up the daily frequencies of four food items (as mentioned above) and expressed in % points (range: 0 to 100). Two various ideas in categorizing diet quality scores (based on data from the cross-sectional study) were applied:

- A posteriori approach—three levels of each diet quality score based on tertile distribution:
  - pHDI: bottom tertile (<20.625% points), middle tertile (20.625–32.125% points), upper tertile (≥32.125% points).
  - nHDI: bottom tertile (<7.875% points), middle tertile (7.875–16.000% points), upper tertile (≥16.000% points).
- A priori approach—three levels of each diet quality score: low (<33.33% points), moderate (33.33–66.66% points), and high (≥66.66% points).

### 2.7. Lifestyle

Four measures were applied to assess lifestyle: screen time, total physical activity, physical activity at school, and leisure time.

Using the question “How much time do you spend watching TV or in front of a computer on an average day of the week?” (Q48; Table S1), screen time (ST) was assessed. The respondents could choose one of six answers (with assigned scores): <2 h/day (0 points), 2 to <4 h/day (1 point), 4 to <6 h/day (2 points), 6 to <8 h/day (3 points), 8 to <10 h/day (4 points), and ≥10 h/day (5 points). Screen time expressed in points was calculated for each participant. The recommendation of the American Academy of Pediatrics of a maximum of two hours of ST per day for children and youth was used as a reference [57]. Three categories of screen time were established after combining some answers: <2 h/day, 2 to 4 h/day, and ≥4 h/day.

Total physical activity (PA) was assessed using two questions regarding physical activity at school and leisure time (Q49–Q50; Table S1). The respondents could choose one of three answers describing their physical activity at school (low, moderate, vigorous) and leisure time (low, moderate, vigorous). Many examples for each answer were given. Finally, after combining some categories of both questions, the respondents were divided into three total physical activity levels: low, moderate and high, with assigned scores from 0 point to 5 points (Table 5). Total PA expressed in points for each participant was calculated. Vigorous PA at school combined with vigorous PA at leisure time (scored with 5 points) was considered as adherence to the World Health Organization recommendation on physical activity [58]. The WHO recommends that children and adolescents aged 5–17 years have a minimum of 2.5 h/day of moderate-intensity PA. Finally, two categories of total physical activity were considered: with the adherence to WHO recommendation on physical activity or no adherence.

### 2.8. Body Composition

The measurements of body weight (kg), height (cm), waist circumference (WC, cm), and hand grip strength (HGS, kg) were taken with a precision of 0.1 kg, 0.1 cm, 0.1 cm, or 0.5 kg, respectively, (between 8 and 12 a.m.) according to the ISAK International Standards for Anthropometric Assessment guidelines [59]. Professional equipment and measuring tape were used, the same type across all the research centers. Table 6 presents the details relating to procedures for body composition measurements. Body mass index (BMI, kg/m^2^) and waist-to-height ratio (WHtR) were calculated. Body mass index was categorized according to age-sex-specific BMI cut-offs for teenagers [60]. A BMI ≥25 kg/m^2^ was used as the overweight measure (general adiposity) and WHtR ≥0.5 as the central obesity measure (central adiposity) [61]. Z-scores of WC, BMI, and WHtR as well as HGS were calculated to achieve mean equal 0 and standard deviation (SD) equal 1. Z-scores were categorized as follows: <−1, −1 to 1 and >1 SD.

### 2.9. Socio-Demographic Data

Respondents were divided into two categories of residence (rural or urban), based on question Q43 (Table S1. The socioeconomic status was determined by using the Family Affluence Scale (FAS). The FAS was a measure of material affluence derived from the characteristics of the household. The scale was developed for the Health Behavior of School-aged Children (HBSC) cross-national study including 44 countries. We used a scale composed of four questions described by the Polish HBSC study team [62]. For each answer, points were assigned (Table S1). Some of the answers were scored with the same number of points.

- Q44: “Does your family own a car, van or truck?” Answers: no (0 points); yes, one (1 point); yes, two or more (2 points).
- Q45: “During the past year, how many times did you travel away on holiday with your family?” (necessary examples were given) Answers: not at all (0 points); once (1 point); twice (2 points); more than twice (2 points).
- Q46: “Do you have your own bedroom for yourself?” Answers: no (0 points); yes (1 point).
- Q47: “How many computers, laptops or tablets do your family own?” Answers: none (0 points); one (1 point); two (2 points); more than two (2 points).

The points were summed up for each respondent (range: 0 to 7). Based on quartile distribution, the respondents were divided into three FAS categories labeled as: low (0–4 points; <25th quartiles), moderate (5–6 points) and high (7 points; ≥75th quartiles).

### 2.10. Sample Size Calculation

Two calculations of minimum sample size were made separately for two study designs.

For the education-based intervention study, the minimum sample size was calculated in regards to nutrition knowledge as a key measure of the study. The calculation was based on the expected increase in nutrition knowledge score after the 9-month follow-up and the expected difference between the educated and control groups. With 5% significance level and 80% power, the sample size required approximately 304 respondents (assuming 2/1 ratio for educated/control group, i.e., 200/104 respondents and an equal number of respondents in each research center) at the end of the study, to detect a 50% difference between the educated group (increase by 30%) and the control group (increase by 15%) in the increase of nutrition knowledge score, including a 50% drop out rate (at the end of the study) and recoding error or missing data of 10%.

For the cross-sectional study, the minimum sample size was calculated in regards to consuming breakfast, which we chose as a key dietary habit and overweight occurrence. The calculation was based on the expected proportion of respondents not consuming breakfast every day (25%) and the difference in overweight occurrence between respondents consuming and not consuming breakfast every day (20% vs. 30%, respectively). With a 5% significance level and 80% power, the minimum sample size required is approximately 904 respondents (assuming a 75/25 ratio for respondents consuming and not consuming breakfast every day, i.e., 678/226 and an equal number of respondents in each research center) to detect a difference between groups in overweight occurrence, including recoding error or missing data of 10%.

### 2.11. Statistical Analysis

Categorical variables are presented as a sample percentage (%), and continuous variables as means with 95% confidence interval (95% confidence interval (CI)) or medians with inter-quartile range for variables with normal or non-normal distribution, respectively. The normality of variables distribution was verified with Kolmogorov-Smirnov test before the statistical analysis. Table 7 presents the general characteristics of the sample across all key variables under study.

A multi-dimensional statistical analysis will be applied to derive dietary-lifestyle patterns (DLPs). We will aim to cluster dietary and lifestyle variables in teenagers in order to identify specific sets of dietary habits existing in combination with lifestyle behaviors (cross-behavioral patterns covering diet and lifestyle). We intend to apply two methods as alternative procedures: Cluster Analysis (CA) and Principal Component Analysis (PCA) [63]. In both analyses, the input variables will be 14 variables (all standardized before) including:

- Eleven dietary, i.e., frequency consumption of breakfast, school meal and nine food items such as dairy products, fish, vegetables, fruit, fruit or vegetable juices, fast foods, sweetened drinks, energy drinks, sweets (in times/day).
- Three lifestyles, i.e., screen time, physical activity at school, physical activity at leisure time (in scores).

A k-means clustering algorithm will be used in CA, and subjects will be grouped into clusters based on the Euclidean distances. The analysis will be conducted several times in order to identify the optimal number of clusters. After selecting clusters, the correctness of cluster identification will be verified by comparing the dietary and lifestyle components of DLPs between clusters with one-way analysis of variance (ANOVA).

A varimax rotation will be used in PCA. The following three criteria will be considered in order to identify the number of PCA-derived patterns: (i) the eigenvalues from the correlation matrix of the standardized variables >1.0, (ii) the break-point identified in the scree plot of the eigenvalues and (iii) the total variance explained. Rotated factor loadings >|0.30| will be considered as significantly contributing to each DLP, interpreted as follows: the higher factor loadings, the stronger association between dietary and lifestyle components of DLPs, and the DLP. For each subject, a DLP score reflecting subject’s adherence to the DLP will be calculated as a sum of the product of the input variables and its factor loadings. In each PCA-derived pattern, subjects will be divided into tertiles based on the DLP scores.

For continuous variables, changes in nutrition knowledge score, Emotional Eating score, Uncontrolled Eating score, Cognitive Restraint of Eating score, pHDI, nHDI, screen time, total physical activity, z-BMI, z-WHtR, z-Waist circumference and z-HGS (all expressed in scores or points or SDs) after 3- or 9-month follow-up in respect to baseline will be verified with ANOVA or Mann-Whitney test, for variables with normal or non-normal distribution, respectively.

For categorical variables, logistic regression modeling will be applied. The odds ratios (ORs) and 95% CIs will be calculated. The significance of ORs will be verified with Wald’s statistics. Crude model and models with an adjustment for confounders will be created.

The following categories of modeled variables in the logistic regression analysis will be included: nutrition knowledge score (low, moderate low, higher), breakfast consumption (skipping, irregular, every day), school meal consumption (skipping, irregular, every school day), food frequency consumption (≤1/week, several times a week, every day), pHDI (tertiles: bottom, middle, upper), nHDI (tertiles: bottom, middle, upper), screen time (<2, 2 to 4, ≥4 h/day), physical activity at school (low, moderate, vigorous), physical activity at leisure time (low, moderate, vigorous), total physical activity (low, moderate, high), CA-derived the DLPs (clusters), PCA-derived the DLPs (tertiles: bottom, middle, upper), BMI (underweight, normal weight, overweight), WHtR (lack of central obesity, central obesity) or z-BMI, z-WHtR, z-Waist circumference, z-HGS (categories for z-scores: <−1, −1 to 1, >1 SD).

The following confounders in the logistic regression analysis will be included: gender, age (years), residence (rural, urban), FAS (points), nutrition knowledge score (points), Emotional Eating score (points), Uncontrolled Eating score (points), Cognitive Restraint of Eating score (points), pHDI (% points), nHDI (% points), screen time (points), and total physical activity (points). For each analysis, the set of confounders will be selected according to the modeled research question.

We will consider assessing four main applications of the logistic regression modeling:

- For the education-based intervention study: (i) a chance to fall in the modeled category after 3- or 9-month follow-up in respect to baseline as reference, (ii) the chance to fall in the modeled category in the educated group in respect to the control group as reference,
- For the cross-sectional study: (iii) adherence to chosen DLP by nutrition knowledge, attitudes toward nutrition and sociodemographic factors in respect to referent DLP, (iv) the chance to fall in the modeled category of body composition in respect to normal body composition category as reference.

All tests will be two-tailed, *p*-values <0.05 will be considered as significant. Analyses will be performed using Statistica software (version 12.0 PL; StatSoft Inc., Tulsa, OK, USA; StatSoft, Krakow, Poland).

## 3. Discussion

This study protocol presents a general approach and details of assessment of nutrition knowledge, attitudes toward nutrition, diet quality, lifestyle, and body composition that were used to comprehensively evaluate the cross-behavioral patterns covering dietary and lifestyle behaviors in teenagers. The protocol also presents a scope of nationwide nutrition education activities taken in Polish teenagers as a part of public health interventions. 

Reducing childhood obesity is not a straightforward task. There are many factors that influence this phenomenon, making it hard to create intervention programs that have a significant impact on obesity reduction. Schools are considered a primary setting for implementing education programs—they have the unique potential to involve a wide population of children and adolescents. However, there are many variables influencing the effectiveness of school-based programs, including school staff who need to be well-trained and familiarized with the program’s aims, procedures, and tools. The creation of education programs by highly-specialized researchers and scientific support for educators at the first stage of implementation of such programs can increase their effectiveness [43,64].

As mentioned before, in Poland a multi-disciplinary approach covering the implementation of multi-component programs are directed mainly to preschool children while relatively few comprehensive educational programs have been combined with scientific assessment of nutrition knowledge, dietary habits, and lifestyle which have targeted teenagers [44,45]. Such programs, focused on reduction of body mass through changing lifestyle and diet have been widely introduced in United States, Australia, China, Brazil, Turkey, and many European countries, e.g., Spain, France, Belgium, Great Britain, Ireland, Germany, Netherlands, Greece, and Norway. Their recapitulation has been presented in several meta-analysis [21,41,65]. There is convincing evidence from this analysis that school-based prevention interventions can lead to an improvement in dietary behaviors by increasing the consumption of healthy foods and decreasing the consumption of unhealthy foods, as well as by changing lifestyle patterns to be more physically active and less sedentary. However, recently published systematic reviews have shown that school-based intervention programs have been at least mildly effective in reducing BMI in children [64,65,66]. It was explained that these new studies tended to have longer observation periods and be more comprehensive, including more factors under study and confounders. Furthermore, more pronounced results were reported in older than younger school children. 

Scientific research shows that linking nutrition knowledge to dietary patterns or diet quality scores would then seem most effective for assessing the relationship between dietary intake and health outcomes. It seems that investing in high-quality research to measure nutrition knowledge is a far-sighted approach, as the burden of diet-related diseases continues to rise worldwide [38,42]. To our knowledge, current intervention and cross-sectional studies should lead to evidence-based initiatives in the field of nutrition education and public health policy. The effectiveness of a public health campaign should be optimized to reduce the prevalence of obesity-related non-communicable diseases, especially in children and adolescents as a vulnerable group.

We anticipate that the results of our comprehensive and multi-factorial statistical analysis will be of great importance. Currently, considering breakfast consumption as a key dietary characteristic along with total physical activity as the key lifestyle characteristic and overweight as the key health outcome, we can discuss these variables based on initial cross-sectional results (in students 11–13 years old). We estimated that 29% of students did not consume breakfast every day. A higher or wider range of the percentage of Polish adolescents (9 to <19 years) not consuming breakfast every day (28–52%) was previously reported [67,68,69,70]. Across Europe, generally more children and adolescents from central (e.g., Slovenia 51–52%) or southern (e.g., Greece 46–48%) than northern countries (e.g., Finland 20%) did not consume breakfast every day [71,72,73]. We found a higher percentage of students (33%) who reached the WHO recommendation on physical activity [58] in comparison with the latest reports (24.2% of Polish children 11–15 years old) [10]. In a further analysis, we will analyze whether this is a positive trend, for example, resulting from the fashion for a healthy lifestyle, or if there are mixed lifestyle behaviors combining greater physical activity with a longer time spent sitting [74]. Overweight was found in 26% of students under study, and this percentage was above the range previously reported in Polish children and young people (12–25%) [5,10]. Thus, our initial findings support previous studies, which have reported that more and more Polish children are overweight or obese.

### 3.1. Strengths and Limitations in General

The main limitation of the study is the use of a questionnaire to collect dietary and lifestyle data. The questionnaire contained a short list of food items and simple questions related to sedentary and active behaviors. However, there is evidence of many advantages of using brief tools, for example, high reproducibility of simple questions, the possibility to rank respondents into categories of habitual food consumption and identify dietary patterns, the possibility to assess compliance with dietary or lifestyle recommendations as well as low-cost, quick and easy administration [75].

The strengths of the study, regarding this questionnaire, are the validation procedure carried out on a large sample of Polish teenagers (630 subjects 11–15 years old). Second, the questionnaire was filled out by teenagers to reduce possible bias, which could be introduced by parents [76]. Future studies should consider the use of a long form of FFQ and/or applying other methods of dietary and lifestyle assessment to fully describe the teenagers’ dietary behaviors and measure physical activity (e.g., use of accelerometry) instead of self-reported data on lifestyle [77,78,79]. However, to date, there is no validated long form of FFQ, which has been developed for Polish children or adolescents.

To measure adiposity, we applied simple obesity measures (BMI, WHtR) with some interpretative limitations but proper for comprehensive assessment of high adiposity levels [60,61,80,81]. In many large epidemiological studies, both measures are widely used. Furthermore, it is easy to interpret results and compare with others due to well-established procedures and strong documented cut-offs developed for children and adolescents [60,61,80]. A fat-free mass was not directly measured, we applied hand grip strength as an indirect measure of muscle mass. There are no cut-offs for the hand grip strength of teenagers, including Polish individuals, so we used z-score of hand grip strength to assess the differences between groups. Future studies should consider the use of more advanced methods of body composition measurement.

To measure socioeconomic status and attitudes toward nutrition, we applied scales dedicated to children (the Family Affluence Scale, FAS, and three-factor eating questionnaire, TFEQ, respectively), both previously validated and used in the Polish population [54,62]. Although we have slightly changed FAS and shortened TFEQ, both new versions have been tested in pilot and validation studies.

Data collection and all measurements were taken by well-trained researchers with the same type of equipment in all scientific centers to minimize inter-center differences. The main study was preceded by a pilot study that was carried out in all centers (covering above 200 subjects).

### 3.2. Strengths and Limitations of the Education-Based Intervention Study

The sample was relatively large (over 460), taking into account that complete dietary and weight status data were obtained at all stages of the study. We applied a very rigorous selection of the sample, each participant had to take part in lectures and workshops within all five topics of education program lasting three weeks—students absent at least one school day for the lectures or workshops were excluded. Next, we used appropriate statistical methods to interpret the differences between the educated and control groups in regards to changes from the baseline to a 3- or 9-month follow-up.

The main limitation is a lack of random subjects to be allocated into the educated and control groups. Unfortunately, we were unable to apply the random approach for several reasons. First, for organizational reasons, we wanted to choose schools located at a convenient distance from the academic centers (up to 50 km). Second (surprisingly), many schoolmasters did not permit their school to participate in the study. Third, many of the schools had previously participated in other nutrition-health education programs, so they could not be included in our study. There was an element of randomness in our study because assigning classes to the educated or control group was accidental and the schools represented a wide social spectrum.

### 3.3. Strengths and Limitations of the Cross-Sectional Study

The main strength of the study was the clustering of dietary and lifestyle variables and identifying sets of dietary habits existing in teenagers in combination with lifestyle behaviors (DLPs). To date, few studies have been aimed at identifying cross-behavioral patterns covering both dietary and lifestyle variables. Although there is limited data available for teenagers from some developed countries [74,76,82,83,84,85,86], there is the gap for Eastern or Central Europe, including Poland.

The main limitation is the sample itself, which was not randomly selected, although it is relatively large (above 1500), has a narrow age range (11–13 years) and covers the entire territory of Poland—widely reflecting the social diversity of Poles (Figure 1A) and providing a good basis for generalizations.

## 4. Conclusions

The study will be able to assess the effectiveness of a nutrition-related and lifestyle-related education program in the medium-term perspective. The weaknesses and strengths of such education programs in respect to teenagers, as a target group, could be identified. Based on the results, in future, well-tailored education programs addressed to teenagers can be created. We think that our results can be interpreted in a wider scope, at least for European teenagers living in similar conditions.

The findings may contribute to identifying teenage subpopulations at risk for excessive body weight and adiposity as well as poor muscling and determine predictors related to diet, lifestyle, nutrition knowledge, attitudes toward nutrition and socio-demographic factors which influence improper body composition and increase obesity risk.

The study provides evidence-based support for preventive health care to promote normal growth and development of the young population and reduce the risk of diet-related diseases in adulthood by the early shaping of adequate dietary and lifestyle behaviors. The results of the study can be implemented as an important public health action.

## Figures and Tables

**Table 1:** Topics and details of the education program.

**Nutrition Topic**	
Goal	Shaping pro-healthy dietary habits.
Scope	Nutrients important in the diet of young people. Health consequences of uncontrolled consumption of energy drinks and dietary supplements. A “Good snack” as an alternative to chips, sticks and sweets. A Pyramid of Healthy Nutrition and Physical Activity [52] and dietary guidelines for teenagers.
Methods	Talk; discussion; workshops.
Activities	Participants propose three various “healthy” breakfasts to take at school by selecting foods from a levels of the Pyramid of Healthy Nutrition and Physical Activity; Participants prepare an “ideal sandwich”.
Tools	Brochure; puzzles; crosswords; website.
**Dietary topic**	
Goal	Supporting well-being, physical, and intellectual development through a healthy lifestyle.
Scope	General recommendations for a healthy lifestyle (healthy eating and physical activity) at school age. The influence of nutrition and physical activity on physical and intellectual development and healthy well-being. The Student Menu—The brain cannot live only on chocolate—A discussion on the most important nutrients in the diet of young people.
Methods	Talk; discussion; workshops.
Activities	Using a pedometer to measure the number of calories consumed during the various activities. Determination of time needed to “burn” the calories contained in the selected product. “Nutrition detective”—measuring the amounts of oil, sugar and salt corresponding to fat, sucrose and salt content in various foods (three sets).
Tools	Brochure; puzzles; crosswords; website.
**Sensory-consumer topic**	
Goal	The world of senses. How to read the food labels? What is important when choosing food?
Scope	Recognition of the basic flavors in aqueous solutions. Discovering the different taste sensations in selected foods. “What kind of a consumer am I”—what do I like and why? Qualification of the consumer’s personality.
Methods	Talk; discussion; workshops.
Activities	Use of sensory memory to identify eight coded odor samples of natural spices, vegetables and fruits. Recognition of selected foods with masked/closed eyes, based on sensory perceptions in the mouth. Preparation of colorful, tasty and healthy snacks from provided foods according to one’s own ideas.
Tools	Brochure; recipes of ‘healthy’ snacks; website.
**Hygiene topic**	
Goal	Food safety. Hygiene during the preparation and consuming of meals.
Scope	The world of microorganisms, pathogens and probiotics. The rules of proper food storage and hygiene during meal preparation and consumption.
Methods	Talk; discussion; workshops.
Activities	Microscopic observation of selected microorganisms—Lactic acid bacteria (*Lactobacillus*) as an example of a microorganism with healthy properties, E. coli (*Escherichia coli*) as an example of a pathogen. Mapping the observed microorganisms’ cells in the prepared templates. Practicing proper hand washing according to instructions. Checking hand cleanness with a test indicator, before and after hand washing.
Tools	Brochure; microscope; test indicator of hand washing; puzzles; crosswords; website.
**Culinary topic**	
Goal	How to prepare healthy, cheap and tasty meals?
Scope	The impact of culinary processes on sensory quality, nutritional value and food safety. The phenomenon of enzymatic browning of fruit and vegetables and ways to prevent this process.
Methods	Talk; discussion; workshops.
Activities	Culinary experiments—preventing the darkening of fruits and vegetables. Checking the impact of storage temperature on the quality of frozen foods, e.g., vegetables. Preparing low-budget healthy meals and low-sweetened beverages.
Tools	A “healthy meals” recipe book; brochure; website.

**Table 2:** The overall content of the education-based intervention study.

Timing	Activities	Group	
		Educated	Control
June 2015 Before education (Baseline)	Data collection with the SF-FQ4PolishChildren questionnaire (nutrition knowledge, attitudes towards nutrition, diet quality, lifestyle, socio-demographic factors).	A	A
	Measurements of body composition (body weight, height, waist circumference, hand grip strength).	A	A
June 2015 Education program (lasting 3 weeks)	The implementation of diet-related and lifestyle-related education program with 5 topics: Nutrition, Dietary, Sensory-consumer, Hygiene, Culinary.	A	NA
June 2015 After education (3 weeks from baseline)	Data collection with the SF-FQ4PolishChildren questionnaire (nutrition knowledge).	A	NA
September 2015 After 3 months from baseline (3-month follow-up)	Data collection with the SF-FQ4PolishChildren questionnaire (nutrition knowledge, attitudes towards nutrition, diet quality, lifestyle, socio-demographic factors).	A	A
March 2016 After 9 months from baseline (9-month follow-up)	Data collection with the SF-FQ4PolishChildren questionnaire (nutrition knowledge, attitudes towards nutrition, diet quality, lifestyle, socio-demographic factors).	A	A
	Measurements of body composition (body weight, height, waist circumference, hand grip strength).	A	A

SF-FQ4PolishChildren—A short form of food frequency questionnaire; A—applied; NA—not applied.

**Table 3:** The overall content of the cross-sectional study.

Timing	Activities	Group	
		Educated	Control
June 2015 Before education	Data collection with the SF-FQ4PolishChildren questionnaire (nutrition knowledge, attitudes towards nutrition, diet quality, lifestyle, socio-demographic factors).	A	A
	Measurements of body composition (body weight, height, waist circumference, hand grip strength).	A	A
June 2016 Before education	Data collection with the SF-FQ4PolishChildren questionnaire (nutrition knowledge, attitudes towards nutrition, diet quality, lifestyle, socio-demographic factors).	A	NA
	Measurements of body composition (body weight, height, waist circumference, hand grip strength).	A	NA

A—applied; NA—not applied.

**Table 4:** School and participant eligibility criteria.

School inclusion criteria: School located at a convenient distance from the academic centers (up to 50 km). The school master permitted their school to participate in the study. The school did not previously participate in other nutrition-health education programs.
Participant inclusion criteria—in general: 4th and 5th grade classes of elementary school. No disability, self-declared by parent or legal guardian or teacher. Written consent of parents or legal guardians to participate.
Participant exclusion criteria—for the education-based intervention study: Data cleaning: age <11 or >12 years, Not attending in all stages of the study with a 9-month follow-up, including all activities of the education program lasting 3 weeks.
Participant exclusion criteria—for the cross-sectional study: Data cleaning: age <11 or >12 years in 2015, and age <11 or >13 years in 2016.

**Table 5:** Categorizing and scoring (points) of total physical activity based on physical activity at school and leisure time.

Physical Activity at School	Physical Activity at Leisure Time		
	Low	Moderate	Vigorous
Low	Low (0)	Low (1)	Moderate (2)
Moderate	Low (1)	Moderate (3)	Moderate (4)
Vigorous	Moderate (2)	Moderate (4)	High (5) Adherence to WHO recommendation

Notes: Physical activity at school: low (most of the time in a sitting position, in class or on breaks), moderate (half the time in a sitting position and half the time in motion), vigorous (most of the time on the move or on classes related to high physical exertion). Physical activity at leisure time: low (more time spent sitting, watching TV, in front of a computer, reading, light housework, a short walk to 2 h a week), moderate (walking, cycling, gymnastics, working at home or other light physical activity performed 2–3 h/week), vigorous (cycling, running, working at home or other sports activities requiring physical effort over 3 h/week).

**Table 6:** Procedures for body composition measurements.

Parameter (Units)	Procedure, Accuracy, and Equipment
Height (H) (cm)	Measurement with the head in horizontal Frankfort plane, Recorded with a precision of 0.1 cm, A portable stadiometer (SECA 220, Hamburg, Germany).
Body weight (BW) (kg)	Measurement in light indoor clothes without shoes, Recorded with a precision of 0.1 kg, Electronic digital scale—the same type across all research centers (SECA 799, Hamburg, Germany).
Waist circumference (WC) (cm)	Measurement at the point midway between the iliac crest and the costal margin (lower rib) on the anterior axillary line in a resting expiratory position, Recorded with a precision of 0.1 cm, A stretch-resistant tape that provides a constant 100 g tension (SECA 201, Hamburg, Germany).
Hand grip strength (HGS) (kg)	Measurement in the standing position, the arm was allowed to move from 180° of flexion to near 0° with maximal effort, Recorded with a precision of 0.5 kg, hydraulic hand dynamometer—the same type across all research centres (SAEHAN Corporation, Masan-Korea-type SH 5001).

**Table 7:** Sample characteristics by study design (% or means (95% confidence interval)).

Parameters	Education-Based Intervention Study (At Baseline)			Cross-Sectional Study		
	Total Sample	Boys	Girls	Total Sample	Boys	Girls
Sample size	464	216	248	1569	760	809
Sample percentage	100.0	46.6	53.4	100.0	48.4	51.6
Age (years)	11.9 (11.9, 12.0)	11.9 (11.9, 12.0)	11.9 (11.9, 11.9)	11.9 (11.9, 12.0)	11.9 (11.9, 12.0)	11.9 (11.9, 12.0)
Age (categories)						
11 years	7.1	5.1	8.9	16.6	15.3	17.9
12 years	92.9	94.9	91.1	73.6	75.9	71.3
13 years	0.0	0.0	0.0	9.8	8.8	10.8
Residence						
rural	34.9	36.1	33.9	40.3	40.3	40.3
urban	65.1	63.9	66.1	59.7	59.7	59.7
Family Affluence Scale (points)	5.3 (5.1, 5.5)	5.2 (4.9, 5.4)	5.4 (5.2, 5.6)	5.4 (5.3, 5.4)	5.3 (5.2, 5.4)	5.4 (5.3, 5.5)
**Nutrition knowledge**						
Nutrition knowledge score (points)	6.0 (5.7, 6.2)	5.5 (5.2, 5.9)	6.3 (6.0, 6.7)	6.1 (5.9, 6.2)	5.7 (5.5, 5.9)	6.5 (6.3, 6.6)
Nutrition knowledge (categories)						
the lowest	30.6	35.6	26.2	30.9	35.7	26.3
moderately low	41.4	42.1	40.7	39.3	40.1	38.6
higher	28.0	22.2	33.1	29.8	24.2	35.1
**Attitudes toward nutrition**						
Emotional Eating (points)	1.3 (1.2, 1.5)	1.3 (1.1, 1.5)	1.4 (1.2, 1.5)	1.3 (1.3, 1.4)	1.3 (1.2, 1.3)	1.4 (1.3, 1.5)
Uncontrolled Eating (points)	5.3 (5.0, 5.5)	5.5 (5.1, 5.9)	5.1 (4.8, 5.4)	5.3 (5.2, 5.4)	5.3 (5.1, 5.5)	5.3 (5.1, 5.5)
Cognitive Restraint of Eating (points)	4.2 (4.0, 4.4)	4.0 (3.7, 4.3)	4.3 (4.1, 4.6)	4.3 (4.2, 4.4)	4.3 (4.1, 4.4)	4.4 (4.2, 4.5)
**Diet quality**						
Every day consumption of						
breakfast	71.0	70.7	71.3	70.0	72.7	67.4
school meal	67.5	59.8	74.2	69.0	64.7	72.9
pHDI (% points)	27.7 (26.4, 29.0)	24.4 (22.8, 26.1)	30.5 (28.6, 32.4)	28.4 (27.7, 29.1)	27.4 (26.3, 28.4)	29.4 (28.4, 30.4)
pHDI (categories)						
low	71.0	81.8	61.7	69.2	72.9	65.8
moderate	28.6	17.8	37.9	29.8	25.9	33.5
high	0.4	0.5	0.4	1.0	1.2	0.7
nHDI (% points)	14.3 (13.3, 15.3)	15.1 (13.8, 16.4)	13.6 (12.2, 15.1)	14.1 (13.5, 14.7)	15.0 (14.1, 15.9)	13.3 (12.5, 14.0)
nHDI (categories)						
low	93.9	94.0	92.7	93.8	93.8	93.8
moderate	6.7	6.0	7.3	5.9	5.5	6.2
high	0.0	0.0	0.0	0.3	0.7	0.0
**Lifestyle**						
Screen time (points)	0.84 (0.74, 0.94)	0.91 (0.76, 1.06)	0.78 (0.65, 0.91)	0.86 (0.81, 0.92)	0.98 (0.89, 1.06)	0.76 (0.69, 0.82)
Screen time (categories)						
<2 h/day	46.5	41.1	51.2	46.3	42.1	50.3
2 to 4 h/day	34.7	40.7	29.4	34.4	35.2	33.6
≥4 h/day	18.8	18.2	19.4	19.3	22.7	16.1
Total physical activity (points)	3.65 (3.53, 3.78)	3.66 (3.48, 3.85)	3.65 (3.49, 3.81)	3.68 (3.61, 3.74)	3.73 (3.63, 3.82)	3.63 (3.54, 3.71)
Total physical activity (categories)						
low	12.3	13.4	11.3	9.8	10.0	9.5
moderate	54.6	49.5	59.1	58.9	53.3	58.9
high	33.0	37.0	29.6	31.3	36.7	26.3
**Body composition**						
Waist circumference (cm)	64.4 (63.5, 65.3)	65.7 (64.3, 67.0)	63.3 (62.2, 64.5)	66.1 (65.6, 66.5)	67.8 (67.1, 68.6)	64.4 (63.8, 65.0)
z-Waist circumference >1 SD	13.5	18.5	9.2	15.1	20.1	10.4
WHtR	0.42 (0.42, 0.43)	0.43 (0.42, 0.44)	0.42 (0.41, 0.42)	0.43 (0.43, 0.43)	0.44 (0.44, 0.45)	0.42 (0.42, 0.42)
Central obesity ^a^	10.0	11.4	8.8	12.2	15.9	8.7
BMI (kg/m^2^)	19.6 (19.2, 19.9)	19.7 (19.1, 20.2)	19.5 (19.0, 20.0)	19.4 (19.2, 19.6)	19.5 (19.2, 19.7)	19.3 (19.1, 19.6)
BMI category ^b^						
underweight	10.2	9.0	11.3	9.8	8.4	11.1
normal weight	63.4	62.3	64.4	65.6	64.8	66.2
overweight	26.4	28.8	24.3	24.7	26.8	22.7
Hand grip strength ^c^ (kg)	20.1 (19.5, 20.8)	20.7 (19.8, 21.6)	19.6 (18.7, 20.5)	20.8 (20.4, 21.1)	21.4 (20.9, 21.8)	20.2 (19.7, 20.6)
z-Hand grip strength ^c^ >1 SD	10.0	11.9	8.3	12.5	14.6	10.4

Sample size may vary in variables due to missing data. pHDI: pro-Healthy Diet Index. nHDI: non-Healthy Diet Index. ^a^ Central obesity identified as waist-to-height ratio ≥0.5 according to Ashwell et al. [61]. ^b^ Body mass index (BMI) categorized according to age-sex-specific cut-offs for teenagers [60]: underweight BMI <18.5 kg/m^2^; normal weight BMI = 18.5 to 24.9 kg/m^2^; overweight BMI ≥25 kg/m^2^. ^c^ Hand grip strength for dominant hand.

## Supplementary Materials

The following are available online at http://www.mdpi.com/2072-6643/10/10/1439/s1, Figure S1: Planned results of the implementation of the national multicenter project ‘ABC of Healthy Eating’, Table S1: The short form of the Food Frequency Questionnaire for Polish Children (SF-FFQ4PolishChildren)—translation into English, Table S2: The short form of the Food Frequency Questionnaire for Polish Children (SF-FFQ4PolishChildren)—Polish language version.
